# Identity Profiles and Well-Being of Multicultural Immigrants: The Case of Canadian Immigrants Living in Quebec

**DOI:** 10.3389/fpsyg.2013.00080

**Published:** 2013-02-27

**Authors:** Joëlle Carpentier, Roxane de la Sablonnière

**Affiliations:** ^1^Département de Psychologie, Université de MontréalMontréal, QC, Canada

**Keywords:** cultural identity, identity integration, well-being, identity profiles, acculturation, self-concept, immigrants

## Abstract

Studies worldwide point toward increased risk of mental health issues among immigrants. Immigrants’ ability to integrate the cultural identity of their new country has been found to be a key factor in their psychological well-being. Even though researchers agree on the crucial role of identity integration in immigrants’ well-being, the current literature has two main limitations: (1) researchers do not agree on the importance that should be allocated to each of the cultural identities, and (2) research has focused on bicultural individuals as opposed to multicultural individuals. The present paper proposes to study Canadians immigrants living in the province of Quebec who, because of the political and linguistic situation of the province, face the challenge of integrating two new cultural identities (Quebecer and Canadian) to their original one. Specifically, cluster analysis was used to observe identity profiles that naturally emerge among 120 Canadian immigrants from the province of Quebec. Identity profiles were then compared on various indices of well-being to identify the optimal identity structure. In total, four identity profiles emerged, differing in their levels of identity coherence (i.e., similar levels of identification with each group) and identification to either the original group or the Quebecers. ANOVA results confirmed that identity profiles differed in their average level of well-being. First, immigrants with coherent profiles displayed higher levels of well-being. Second, among incoherent profiles, the profile where identification to the original group is the highest showed the greatest well-being. Implications suggest that in order to maximize immigrants’ well-being, psychologists should focus on the coherence between cultural groups as well as identification to the original group.

## Introduction

With the advent of globalization, the borders between countries have fallen, resulting in individuals being increasingly likely to leave their homeland to find work, safety for their family, or even happiness somewhere else. Recent surveys have estimated to 191 million the total number of people across the world living in a country different from the one they were born in (Van Oudenhoven et al., 2006).

Even though most immigrants move to a new country with the hope of a better life, their new reality comes with its share of challenges. Past studies, for example, showed that immigrants’ psychological well-being and mental health often suffer from the enormous change that migration represents. Indeed, studies have highlighted the increased risk of psychotic disorder among immigrants (Zolkowska et al., 2001; Cantor-Graae and Selten, 2005; Cantor-Graae et al., 2005). Other studies have also shown that immigrants display higher rates of anxiety and depressive disorders than non-immigrants (Carta et al., 1991; Esteban y Pena, 2001).

A pivotal element in the immigrants’ adaptation process is identity (Lee, 2003, 2005; Wong et al., 2003; Walsh et al., 2012). Specifically, immigrants’ ability to integrate the cultural identity of their country of origin and the cultural identity of their new country to form a harmonious self-concept has been found to be positively related to various forms of psychological well-being (Crocker et al., 1994; Cameron, 1999; Roberts et al., 1999; Benet-Martinez et al., 2002; Roccas and Brewer, 2002; Downie et al., 2004;  Amiot et al., 2007). Reduced personal uncertainty (Hogg and Mullin, 1999; Hogg et al., 2007; Usborne and Taylor, 2012), diminished internal conflicts (Benet-Martinez et al., 2002), or greater collective support (Côté and Levine, 2002;  Schwartz et al., 2006) are among the potential mechanisms that could explain the positive relation between identity integration and well-being.

Even though researchers agree on the crucial role of identity integration in immigrants’ well-being, the current literature on identity integration has two main limitations. First, the importance that should be allocated to each of the cultural identities (i.e., cultural identity of the country of origin and cultural identity of the new country) in the overall self-concept in order to maximize immigrants’ psychological well-being remains unclear. Indeed, there is no consensus among researchers regarding the optimal integrated identity structure. While some researchers argue that a strong identification to both the original and the new groups maximizes well-being (Berry, 1997), others insist more on the importance of maintaining a coherent identity, regardless of the strength of identification (Amiot et al., 2007). Finally, some authors put the emphasis on identifying to an inclusive group where both the culture of origin and the new culture are included (Gaertner et al., 1993; Hornsey and Hogg, 2000a,b,c). The second limitation is that, to our knowledge, most researchers have focused on bicultural individuals. Therefore, little is known on the identity structure that maximizes the well-being of immigrants, especially among those having more than two cultural identities.

The present study proposes to overcome these limitations by examining identity profiles that naturally emerge among Canadian immigrants living in the province of Quebec, and by comparing these profiles on their average level of psychological well-being, in order to identify the identity structure that maximizes their well-being. To do so, the context of Quebec immigration will first be presented, followed by a discussion on the main theoretical models dealing with immigrants’ identity structure and its impact on well-being.

### The context of Quebec immigration

With almost 20% of the total Canadian population being foreign-born (Gouvernement du Québec, 2009), Canada is one of the countries with the highest percentage of immigrants among its total population. Even though Canada comprises 10 provinces and 3 territories, almost 14% of Canadian immigrants choose to settle in the province of Quebec (Gouvernement du Québec, 2009), which has a distinctive political and cultural situation. Indeed, the province of Quebec is the only province in Canada where French is the only official language, with 80% of Quebec residents being Francophone (Gouvernement du Québec, 2009). Conflicts between the Anglophone and Francophone communities characterized the province of Quebec for more than two centuries, and still do today. On the one hand, Francophones have faced economic disadvantages and threats to their language and culture throughout their history, mainly because of their minority status in an English-dominated country and continent (Bougie et al., 2011). As a result, they have developed a strong desire to protect and preserve their distinctiveness. Anglophones, on the other hand, used to be an elite minority in Quebec. However, they suffered from the growth of Francophone nationalism in the past 50 years, which resulted in a reversal of power between Francophones and Anglophones and have left the latter feeling increasingly threatened (Caldwell, 1984; Bourhis, 1994). Quebec history was marked by two referendums on Quebec sovereignty in 1980 and 1995. The results of the last one were very close, with the motion to decide whether Quebec should secede from Canada being defeated by a very narrow margin of 49.42% “Yes” to 50.58% “No.”

To become a Canadian citizen, one must succeed at a citizenship test in which the understanding of the rights, responsibilities, and privileges of citizenship, as well as of Canada’s history, values, institutions, and symbols are evaluated. In addition, one must demonstrate adequate knowledge of either French or English (Minister of Citizenship and Immigration Canada, 2012). However, since Canada uses a points-based system to determine who can come in as an immigrant, people wishing to migrate specifically to Quebec are significantly advantaged when they master French, since an important number of points is awarded to the mastery of this language, compared to English, for this specific province (http://www.immigration-quebec.gouv.qc.ca/fr/informations/note-competences-linguistiques.html).

In sum, because of the particular linguistic situation of the province, there are two main cultural identities in Quebec: the “Quebecer”^1^ (associated with the Francophone culture) and the “Canadian” (associated with the Anglophone culture). Immigrants arriving in Quebec thus face a particular challenge. They bring with them the cultural background of their country of origin, they are officially greeted by the country of Canada and must learn the basics of the Canadian history and culture to have their Canadian citizenship, but once they arrive to Quebec, they are also confronted in their day-to-day interactions with the Quebecer culture and the necessity to learn the French language. Immigrants moving to Quebec thus have to integrate two new cultural identities in their global self-concept.

Thus, we argue that finding the identity structure that maximizes the well-being of multicultural immigrants is particularly relevant for Canadian immigrants living in the province of Quebec. In addition, with almost half (46.6%) of Quebec total population growth between 2001 and 2006 being due to the increase in the immigrant population during this period (Gouvernement du Québec, 2009), the Quebec as a whole would benefit from increased levels of well-being among its immigrants.

In order to draw a complete picture of various identity structures adopted by immigrants, and then better understand their potential impacts on well-being, the acculturation model, the cognitive-development model of social identities integration and the common ingroup identity model are reviewed next.

### Acculturation model

The acculturation model focuses on the strategy adopted by immigrants to simultaneously approach their culture of origin and the new culture (Berry, 1990, 2003; Sam, 2006). It proposes to independently assess the extent to which immigrants maintain their heritage culture (i.e., identification to the original cultural group) as well as the degree to which they seek involvement with the culture of the society of settlement (i.e., identification to the new cultural group). Crossing these two dimensions, Berry (1997) proposes four strategies of acculturation. Immigrants adopting an *assimilation* strategy do not preserve their identification to their original cultural group and choose to identify with the new culture. At the opposite, the *separation* strategy refers to immigrants choosing to maintain their original cultural identity while avoiding identification with the new culture. The third strategy, named *marginalization*, refers to immigrants seeking neither cultural maintenance nor identification with the new culture. Finally, the *integration* strategy is defined as strongly identifying with both the original cultural group and the cultural group of the society of settlement.

Studies on the acculturation model have shown that the *integration* strategy is the one that maximizes immigrants’ long-term well-being (Krishnan and Berry, 1992; Schmitz, 1992; Sam and Berry, 1995; Berry, 1997; Phinney and Devich-Navarro, 1997). However, the reality of Canadian immigrants living in the province of Quebec makes the two axes, i.e., identification to the new culture and to the original cultural group, insufficient to accurately reflect people’s organization of cultural identities. As an example, we can think about a Russian woman migrating to Canada with her family and choosing to live in Quebec. The acculturation model does not allow to answer questions such as: In order to maximize her well-being, does the woman need to strongly identify with both the Canadian and the Quebecer cultures? Should the Canadian or Quebecer cultures be as important for her as the Russian culture? Is it possible for her to strongly identify with all of her cultural groups at the same time? One of the main limitations of the acculturation model is thus that it cannot directly inform us on the identity structure that maximizes the well-being of immigrants having to deal with more than two cultural identities.

### Cognitive-development model of social identities integration

The cognitive-development model of social identity integration, proposed by Amiot et al. (2007), is a dynamic model that conceptualizes the process by which social identities become integrated into the self-concept. The authors do not focus on two specific identities, as it is the case with the acculturation model, but instead attempt to draw a complete picture of the multiple social groups to which an individual belongs.

This model considers that multiple identities are integrated into the self-concept when they can be simultaneously and equally important for the individual (Roccas and Brewer, 2002; Amiot et al., 2007). An individual with an integrated self-structure has used various strategies to resolve conflicts that might have existed between identities, and now equally identifies to each of his/her groups of belonging, while also differentiating each of them in his/her global self-concept (e.g., Donahue et al., 1993; Rafaeli-Mor and Steinberg, 2002). Identity integration is thus reached, and well-being maximized, when coherence is achieved. While coherence usually refers to the extent to which two identities are interrelated or associated to one another (Donahue et al., 1993), Amiot and her colleagues refer to coherence as the fact to confer equal importance to all groups of belonging (Amiot et al., 2007; de la Sablonnière et al., 2010). Although these might seem like two distinct definitions, they are rather complementary. Indeed, in the cognitive-development model of social identity integration, it is argued that the more two identities are associated, the more they will be perceived as similar and consequently, the more the person should equally identify to every group. Equal level of identification to every group is thus seen as an indicator of coherence and of identity integration.

Based on this model, the Russian woman from our example should equally identify with the Russian, the Quebecer and the Canadian cultures to achieve identity integration, and ultimately well-being. If the Russian woman fails to integrate her various identities into a coherent self-concept, she might exclusively define herself with one of her cultures (categorization, Amiot et al., 2007). She might also tend to feel Russian in certain situations and Canadian or Quebecer in others (compartmentalization, Amiot et al., 2007). In a case where there is little coherence between identities, the Russian woman would constantly feel torn between the different cultural groups to which she belongs, which would consequently impede her well-being.

The cognitive-development model of social identity integration, which puts the emphasis on coherence between the forces of identification rather than on the strength of identification itself, generates one important criticism: it implies that it is as beneficial to identify weakly with all groups as it is to identify strongly with all of them. This statement goes against the acculturation model that argued that the strengths of identification to the cultural groups are relevant for immigrants’ well-being (Krishnan and Berry, 1992; Schmitz, 1992; Sam and Berry, 1995; Berry, 1997; Phinney and Devich-Navarro, 1997). However, past research comparing Berry’s acculturation model and the cognitive-development model suggests that the marginalization strategy (low identification to new and original cultural groups) might indeed be as beneficial for well-being as the integration strategy (high identification to both cultures; de la Sablonnière et al., 2010). More importantly, that research clearly demonstrated the stronger predictive power of the cognitive-development model of social identity integration, as compared to Berry’s acculturation model, when predicting well-being. Such conclusion, however, remains to be replicated.

### Common ingroup identity model

The common ingroup identity model (Gaertner et al., 1993) assumes a hierarchical organization in the self-concept of the various groups to which a person belongs. Groups located at higher levels are considered more inclusive, which means that they gather several cultural groups from lower levels. The “Europeans” would for example be considered of higher level than the “Italians” because this first group can include cultures from different countries. The “Italians,” in turn, refer to a group of higher level than the regional cultures because it includes, for example, the “Sicilian” and the “Sardinian” cultures.

According to the common ingroup identity model, individuals facing the challenge of integrating multiple identities into their self-concept should identify with a more inclusive group that comprises all of the groups they belong to. For instance, the woman in our example could define herself as “Canadian,” a culture that includes people of various cultural backgrounds who all share the fact that they now live in Canada, while also including the cultures of its provinces such as Quebec. Such recategorization of the “us” and “them” into a “we” should result in a decreased salience of the differences between groups. Identifying to a more inclusive group should thus be beneficial for one’s well-being as it reduces internal and external conflicts between identities.

However, researchers do not entirely support this model. Hornsey and Hogg (2000a,b,c) showed that identifying to a more inclusive group is beneficial for one’s well-being only if identification to the original group can be maintained. In this case, it would be beneficial for the Russian woman to define herself as Canadian only if she feels that the Russian and Quebecer cultures are welcomed within (and are not supplanted by) the Canadian culture. The cognitive-development model of social identity integration (Amiot et al., 2007) also addresses the importance of identifying to a more inclusive group, but considers it as a step in the identity integration process rather than an end in itself (Amiot et al., 2007). Indeed, according to Amiot et al. (2007), identification with a more inclusive group would contribute to establish links between various cultural identities and thereby help achieving a coherent identity.

### The present research

Considering the contrasting predictions from the acculturation model, the cognitive-development model, and the common ingroup identity model, it becomes pivotal to compare these theories in order to fully comprehend how a Canadian immigrant living in the province of Quebec can organize his/her cultural identities in order to maximize his/her well-being. To do so, we will examine identity profiles that naturally emerge among them. Using clusters analysis, immigrants who organize similarly their identities will be grouped together to create what we labeled *identity profiles*. These profiles will contribute to the identification of the identity structure that maximizes the well-being of Canadian immigrants living in Quebec in many ways. First, they will offer a predictive model that includes all the concepts proposed in other main theories. Indeed, identity profiles will include the strength of identification to both the original and the new (Quebecers) cultural groups, while also addressing identification to a more inclusive group (Canadians) as well as coherence between these three groups. Second, when testing the link between identity profiles belonging and well-being indicators, it will be possible to compare the existing theories by looking at the characteristics (i.e., strength of identification to each group, coherence between identities and impact of the dominant identity) of the profile that maximizes well-being. Third, identity profiles will allow overcoming an important limitation of some existing theories on identity integration by considering identification to more than two cultural groups at the same time. Finally, the relevance of using identity profiles to predict immigrants’ well-being will be tested by comparing their predictive power to the one of well-established concepts in the literature, namely perceived distance and conflicts between cultural identities (Benet-Martinez et al., 2002). Past studies with bicultural individuals have indeed shown that the more people perceive their cultural groups as distant and conflictual, the lower their level of well-being is (Nguyen and Benet-Martinez, 2007). We postulate that, for multicultural immigrants, using identity profiles to look at their identity structure will be more relevant to predict their well-being than examining their perception of distance and conflicts between their identities. This will be further addressed in the sub-section “additional analyses” of the results section.

Using identity profiles and based on the main theoretical models presented above, four main hypotheses are presented. However, only two of these hypotheses are expected to be testable within the specific context of Canadian immigrants living in the province of Quebec.

Based on the cognitive-development model of social identity integration proposed by Amiot et al. (2007), we first hypothesize that:

H1: Coherent profiles (i.e., immigrants equally identifying with the Quebecers, the Canadians, and to their original group) will display significant higher levels of well-being than incoherent profiles.

Based on the acculturation model (Berry, 1997, 2006), it is also postulated that:

H2: Among coherent profiles, profiles where people identify more strongly with the Quebecers, the Canadians and with their original group will display significant higher levels of well-being as compared with profiles with lower levels of identification.

However, considering that past studies on Berry’s acculturation model have always found that only few people are marginalized (i.e., low identification to all groups of belonging), combined to the fact that our participants were recruited in advanced French class which probably facilitate their integration within the Quebecer and Canadian cultures, it would be surprising to find a coherent profile with low identification to the three groups. Hypothesis 2 might therefore not be testable with our sample.

Finally, hypotheses concerning the impact of the dominant identity among incoherent profiles are also postulated. It is first important to mention that the “Canadian” identity will be considered the most inclusive cultural identity in our study. Indeed, the “Canadian” cultural identity can be considered more inclusive than the “Quebecer” and the “Original group” identities. First, using a hierarchical perspective, the “Canadian” identity is a national identity and thereby includes the identities of its provinces (such as Quebec) as well as identities of its specific cultural groups (such as immigrants). Second, its content and meaning is also inclusive. Indeed, with almost 20% of the Canadian population aged 18 years and older being first or second generation immigrants in 2004 (Fleury, 2007), Canada is considered a multicultural country. As such, the “Canadian” cultural identity includes ideas, values, beliefs, and knowledge that characterize Canadians as diverse and accepting of cultural minorities. Even though the province of Quebec welcomes an important proportion of Canadian immigrants, Quebecers are not considered as accepting toward other cultures as Canadians are (Presse Canadienne/Léger Marketing, 2001; Helly, 2004; Amiot and de la Sablonnière, 2010). In short, the Canadian identity can be considered more inclusive than the Quebecer identity both from a hierarchical perspective and in terms of its content.

Considering that the current state of knowledge does not allow us to decide which cultural group will have the greatest impact on well-being when dominant over the others, two contrasting hypotheses are postulated. First, based on the common ingroup identity model (Gaertner et al., 1993), we hypothesize that:

H3a: Among incoherent profiles, the ones where individuals identify strongly with the most inclusive group (i.e., the Canadians) will display significant higher levels of well-being than the ones where the dominant identity is not inclusive (i.e., Quebecer or original group).

Second, based on past research that highlighted the crucial role of the maintenance of the identification to the original group (Hornsey and Hogg, 2000a,b,c), this second contrasting hypothesis is postulated:

H3b: Among incoherent profiles, the ones where individuals identify strongly with their original group will display significant higher levels of well-being than other incoherent profiles.

Even though the two contrasting hypotheses are postulated, hypothesis 3b is expected not to be testable in our sample. More specifically, we expect to find no profiles in which people strongly identify with the Canadian culture without also strongly identifying with the Quebecer culture. Our expectations are based on the fact that the Francophone culture (called the Quebecer culture in our study) is crucial in distinguishing the province of Quebec from other Canadian provinces and territories. As described in the section on the context of Quebec immigration, the cultural, and linguistic situation of the province of Quebec is so particular that we believe that people wishing to migrate to Quebec have specific characteristics such as demonstrating knowledge of French and having an interest for Quebec prior to their arrival. We therefore believe that it would be surprising to find immigrants that strongly identify with Canadians without also strongly identifying with Quebecers. In addition, it is expected that most of the immigrants who arrived in the province of Quebec and did not identify with the Quebecer culture, while strongly identifying to the Canadian culture, would eventually have moved to another province. Indeed, learning French and identifying with the Quebecer culture significantly facilitate crucial elements such as employment, housing, and social services, especially outside Quebec’s main city (e.g., Montreal). Finally, immigrants who are still living in Quebec while identifying strongly with the Canadians but not the Quebecers would probably not be interested in following an advanced French class, which is where our participants were recruited.

## Materials and Methods

### Participants

A total of 120 participants (74 women and 46 men) took part in the present study. When asked to indicate their country of origin, 41% of them indicated a country within Eastern Europe or Russia, 20% a country of the Middle East, 17% were from Latin America or the Caribbean, 13% from Asia, 3% from a country within Western Europe, 3% from a North African country, 1% from a country within the rest of Africa, and 2% did not specified their country of origin. Participants were aged between 17 and 52 years old (*M* = 30.00) and had been living in Canada for 4.42 years in average (SD = 6.38).

### Procedure

Administrators of establishments offering French classes to new immigrants in the province of Quebec were solicited by phone and email. These centers aim to develop oral and written French skills for new immigrants whose native language is not French, in order to prepare them for further studies or for the labor market. Following the obtainment of administrators’ and teachers’ consent, students from the advanced level were met during a class. Since the questionnaire was in French, only immigrants taking part in the advanced classes were met to ensure they mastered French sufficiently well to understand the questions being asked. Instruments were translated in French using the back-translation procedure (Brislin, 1970; Vallerand, 1989). It was explained that the goal of the study was to better understand the way immigrants define themselves and that participation to the study was entirely voluntary.

### Measures

Participants filled out a questionnaire including measures of identification to three cultural groups (the Canadians, the Quebecers, and their original group) as well as measures of collective and individual well-being. Participants were asked to indicate at the beginning of the questionnaire the cultural group that they considered their “original cultural group,” apart from the Quebecers or the Canadians. They were then told that every time they would see the expression “your original cultural group,” they would have to think about this specific group. This methodology was chosen because it allowed us to make no assumption regarding our participants’ cultural background. Some participants (13% of the sample) indicated groups that could actually include multiple cultural groups, such as “Israeli-Russian” or “Latinos,” while the rest of the answers were cultural groups that were directly in line with participants’ nationalities (e.g., Chinese or German).

Items related to identification to the cultural groups and to collective well-being were asked three times, once for each cultural group. Since the same questions were always asked three times, four different versions of the questionnaire were distributed, counterbalancing the order of the questions. Analyses confirmed that results were not affected by the order in which the questions were asked. All responses will thus be kept as a single group in our main analyses. Demographic variables such as age, gender, nationality, and the number of months since their arrival in Canada were also included in the questionnaire.

#### Identification to cultural groups

Five-items were used to assess participants’ cognitive identification with the three groups (Ellemers et al., 1999; Jackson, 2002). Examples of items are “I identify with Canadians,” “I identify with Quebecers,” and “I identify with members of my original group.” Participants were asked to indicate the extent to which they agreed with each statement using an 11-point scale ranging from “Do not agree at all” (0) to “Totally agree” (10). This scale showed good reliability in the present study (Quebecer: α = 0.82; Canadian: α = 0.77; original group: α = 0.81).

#### Collective well-being

Collective well-being was evaluated using two different measures in order to ensure the stability of our results.

##### Collective esteem

Collective esteem represents the *evaluative* component of social identity and can be defined as the part of self-esteem that arises from group membership (Taylor, 1997, 2002). It has been used as an indicator of collective well-being in numerous previous studies (e.g., Branscombe et al., 1999; Walker, 1999). Five-items were used to assess participants’ feeling of collective esteem. Based on Ellemers et al. (1999) and Jackson (2002), participants evaluated how positive they felt as members of each group using an 11-point response scale ranging from “Do not agree at all” (0) to “Totally agree” (10). An example of item for this scale is “I am glad I am Canadian” (or Quebecer, or a member of my original group; Quebecer: α = 0.83; Canadian: α = 0.83; original group: α = 0.84).

##### Collective hope

Collective hope represents the hope in the betterment of one’s group. In the present study, collective hope was assessed using a three-item scale inspired from a scale assessing personal hope (Snyder et al., 1996). The collective application of the personal hope scale has been shown to be a reliable indicator of collective well-being in the past (de la Sablonnière et al., 2009). Participants indicated, using an 11-point response scale ranging from “Do not agree at all” (0) to “Totally agree” (10), the extent to which they agreed with each item. A sample item is “I think that Quebecers’ (or Canadians’, or my original group’s) situation will get better in the future.” This scale showed good reliability in the present study (Quebecer: α = 0.85; Canadian: α = 0.82; original group: α = 0.84).

#### Individual well-being

Individual well-being was assessed using the self-esteem, life satisfaction, and subjective vitality scales.

##### Self-esteem

The Rosenberg’s Self-Esteem Scale (Rosenberg, 1965) assesses people’s global perception of their self-worth and their general sense of self-acceptance. A shortened five-item version of the scale was used in the present study (α = 0.79). Participants indicated the extent to which they agreed with each item using an 11-point response scale ranging from “Do not agree at all” (0) to “Totally agree” (10). A sample item is: “I feel that I am a person of worth, at least on an equal basis with others.”

##### Life satisfaction

The Satisfaction with Life Scale (Diener et al., 1985; Blais et al., 1989) was used to evaluate participants’ life satisfaction (sample item: “I am satisfied with my life,” α = 0.74). This five-item scale assesses participants’ level of satisfaction with their life in general using an 11-point Likert-type response scale ranging from “Do not agree at all” (0) to “Totally agree” (10).

##### Subjective vitality

The Subjective Vitality Scale (Ryan and Frederick, 1997) was used to evaluate the extent to which participants generally felt alive and energetic. Using five-items and an 11-point Likert-type response scale ranging from “Do not agree at all” (0) to “Totally agree” (10), participants indicated the extent to which they agreed with statements such as “I look forward to each new day.” This scale showed acceptable reliability in the present study (α = 0.66).

#### Perceived distance and conflicts between identities

A shortened five-item version of the Bicultural Identity Integration (BII) Scale (Benet-Martínez and Haritatos, 2005; Huynh, 2010) was included in the questionnaire to assess perceived cultural conflict (three items, α = 0.63) and cultural distance (two items, *r* = 0.68, *p* < 0.001) between the Quebecer and original group cultures, as well as between the Canadian and original group cultures (conflict, α = 0.72; distance, *r* = 0.71, *p* < 0.001).

## Results

### Preliminary analyses

Preliminary analyses showed that the number of missing values was less than 2% for all variables, except for indicators of individual well-being (i.e., self-esteem, life satisfaction, subjective vitality) where each had 5.83% of missing values. Since items related to individual well-being were at the end of the questionnaire and that some participants did not have sufficient time to finish the questionnaire within the period allocated to it during class, the number of missing values is not surprising. Considering that the proportion of missing values is close to the 5% limit fixed by Tabachnick and Fidell (2007) and that excluding these participants did not change the results, we decided to keep them in the analyses in order to maximize sample size when creating clusters. The database was then inspected to detect the presence of multivariate and univariate outliers. Five participants were removed from further analyses because they exceeded the critical χ^2^ value of 34.52 (*p* < 0.001) and could therefore be considered mutltivariate outliers.

Means, standard deviations, and correlations between all key variables, as well as indicators of skewness and kurtosis are presented in Table 1. All variables were normally distributed, as indicated by skewness and kurtosis scores ranging from −0.97 to 1.23 (Kline, 2005).

**Table 1:** **Descriptive statistics and correlations**.

Variables	Correlations											
	1	2	3	4	5	6	7	8	9	10	11	12
1. Identification to Quebecers	–	0.51***	0.10	0.70***	0.38***	0.32***	0.20*	−0.11	0.01	0.10	0.15	0.02
2. Identification to Canadians		–	0.10	0.43***	0.25**	0.69***	0.41***	−0.04	−0.01	0.14	0.18	0.19*
3. Identification to original group			–	0.03	0.21*	0.05	0.16	0.68***	0.37***	0.25*	0.10	0.12
4. Collective esteem – Quebecers				–	0.43***	0.54***	0.24**	−0.07	−0.11	0.08	0.14	0.10
5. Collective hope – Quebecers					–	0.14	0.65***	0.12	0.13	0.28**	0.24*	0.32***
6. Collective esteem Canadians						–	0.35***	0.03	−0.07	0.12	0.14	0.24*
7. Collective hope – Canadians							–	0.13	0.15	0.31***	0.20*	0.31***
8. Collective esteem– original group								–	0.52***	0.20*	0.18	0.23*
9. Collective hope – original group									–	0.28**	0.16	0.24*
10. Self-esteem										–	0.60***	0.62***
11. Life satisfaction											–	0.49***
12. Subjective vitality												–
*N*	115	114	114	115	115	115	115	114	113	108	108	108
Mean	6.02	6.30	6.80	6.95	7.06	7.53	7.50	7.34	6.82	8.13	6.91	7.12
SD	1.91	1.69	2.01	1.85	1.83	1.82	1.49	1.96	2.04	1.44	1.57	1.55
Skewness	−0.08	−0.11	−0.78	−0.48	−0.29	−0.66	−0.14	−0.97	−0.83	−0.96	−0.27	−0.06
Kurtosis	−0.78	−0.59	0.13	−0.08	−0.23	−0.23	−0.73	1.23	0.61	−0.54	−0.53	−0.37

*p < 0.05, **p < 0.01, ***p < 0.001.

### Main analyses

#### Identity profiles

The first step in testing our hypotheses was to examine identity profiles that naturally emerge among Canadian immigrants living in Quebec. To identify sub-groups of immigrants based on their identity profiles, a cluster analysis was conducted. Cluster analysis is a statistical method that identifies sub-groups within the sample that show similar characteristics. The clustering variables were the levels of identification with the Quebecers, Canadians, and the original cultural group. Raw scores were used because all variables shared the same response scale. Considering that it was impossible to theoretically determine *a priori* the number of clusters that should be uncovered, a hierarchical cluster analysis using Ward’s linkage method with the squared Euclidian distance measure was performed to obtain the solution that best represented the data in the sample. Hierarchical cluster analysis is an exploratory data reduction technique that gradually combines observations between which the distance is not statistically different from 0 (Burns and Burns, 2008; Mooi and Sarstedt, 2011). It starts with each case as a separate cluster and then combines the two participants showing the shortest distance. In the next step, a third participant, which has the next least distance, is added to the cluster to create a three-observation cluster. The algorithm continues until all the observations are in one cluster. With the Ward’s linkage method, the criterion for fusion of observations is that it should produce the smallest possible increase in the error sum of squares when calculating the total sum of squared deviations from the mean of a cluster (Burns and Burns, 2008; Mooi and Sarstedt, 2011).

Cluster analysis allows researchers to examine different solutions, and then select the solution that best fits the data (Hodge and Petlichkoff, 2000; Cumming et al., 2002). The agglomeration coefficient and dendrograms suggested that a four-cluster solution was the most appropriate.

**Figure 1:**
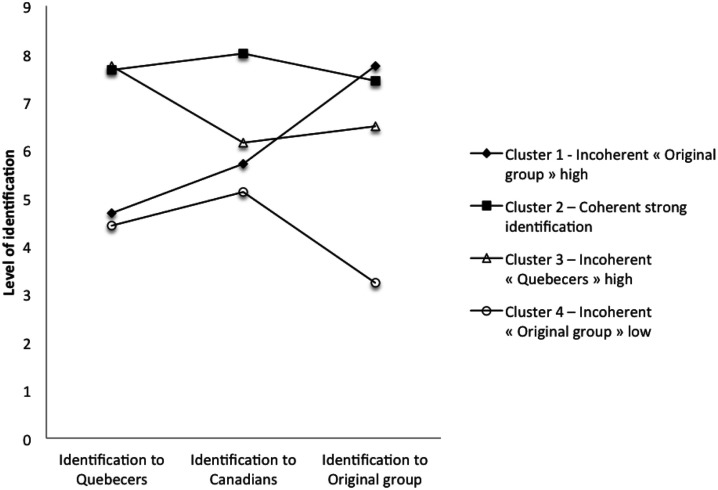
**Level of identification to cultural groups as a function of clusters**.

Means of the levels of identification to each cultural group for the four-cluster solution are reported in Table 2. Figure 1 displays the identification levels as a function of clusters. In order to identify what differentiates one cluster from another, a one-way multivariate analysis of variance (MANOVA) was conducted with the levels of identification to the three cultural groups as dependent variables and the four clusters as the independent variables. Results revealed significant differences among the four groups, *F*(9, 243.52) = 37.41, *p* < 0.001, Wilks’ Λ = 0.12. A one-way ANOVA was conducted on each dependent variable as a follow-up to the MANOVA. To protect from the inflation of type I error probabilities that occurs with multiple testing, a Bonferroni correction was applied and the acceptable level of significance was fixed at *p* = 0.013. The ANOVAs revealed that clusters significantly differed on levels of identification with Quebecers, *F*(3, 102) = 59.50, *p* < 0.001, with Canadians, *F*(3, 102) = 16.13, *p* < 0.001, and with the original group, *F*(3, 102) = 44.81, *p* < 0.001. Table 2 shows the complete picture of all significant differences among the four clusters. Overall, these differences support the idea that the four clusters are distinct from one another.

**Table 2:** **Means for the clustering variables as a function of clusters**.

Clusters	Cluster 1 – incoherent « original group » high	Cluster 2 – coherent strong identification	Cluster 3 – incoherent « Quebecers » high	Cluster 4 – incoherent « original group » low	*F*	*p*
Identification to Quebecers	4.68_a_	7.66_b_	7.74_b_	4.42_a_	59.50	0.00
Identification to Canadians	5.71_a_	8.00_b_	6.15_a_	5.12_a_	16.13	0.00
Identification to Original group	7.74_a_	7.44_a,b_	6.50_b_	3.23_c_	44.81	0.00
*N*	47	20	25	14		

*For each dependent variable, means with different subscripts indicate a significant difference at *p* < 0.01 using Scheffé’s *post hoc* test*.

Identification levels to the groups, and coherence between them, allow us to label the four clusters. Participants in the first cluster represented 44% of the sample (*n* = 47) and included immigrants who identified mildly with Quebecers and Canadians but strongly identified with their original group, thereby showing incoherence between identification to the various groups. This cluster was labeled “Incoherent – Original group high.” The second cluster represented 19% of the sample (*n* = 20) and included immigrants whose identity profile was characterized by high levels of identification to the three groups and consequently by a high level of coherence. This second cluster was thus labeled “Coherent – Strong identification.” The third cluster represented 24% of the sample (*n* = 25) and included immigrants who identified mildly with Canadians and their original group, but strongly identified to Quebecers. This third cluster was labeled “Incoherent – Quebecers high.” Finally, the fourth cluster represented 13% of the sample (*n* = 14) and included participants who displayed moderate levels of identification to Quebecers and Canadians, while identifying even less with their original group. This cluster was labeled “Incoherent – Original group low.”

For exploratory purposes, we then compared the four clusters to determine if their members differed on demographic variables such as their country of origin and the number of years they had been living in Canada. Participants’ countries of origin were coded in seven categories: (1) Eastern Europe and Russia, (2) Western Europe, (3) Northern Africa, (4) Africa, (5) Eastern Asia, (6) Latin America and the Caribbean, and (7) Middle East. A chi-square test of independence first allowed testing if belonging to one category (country of origin) was related to belonging to a specific cluster. Results showed that there was no significant relationship between the country of origin and the identity profile (χ^2^ = 17.41, *df* = 15, *p* = 0.30). Second, a one-way ANOVA revealed that clusters did not significantly differ in regard to the average time since the arrival in Canada of their members, *F*(3, 100) = 0.65, *p* = 0.58. Finally, interaction effects were also tested to see if the number of years since arrival in Canada or the country of origin could moderate the impact of identity profiles on well-being. Since no significant interactions effects were found, we decided not to include these analyses in the present paper.

#### Identity profiles differences in well-being

The main goal of this study was to determine which identity structure maximizes the well-being of Canadian immigrants living in Quebec. Three steps in analyzing the results were performed. First, two one-way MANOVAs were conducted, one with the six indicators of collective well-being (two indicators for each of the three cultural groups) as dependent variables and one with the three indicators of individual well-being as dependent variables. Results first revealed significant differences among the four profiles for collective well-being, *F*(18, 294.00) = 7.10, *p* < 0.001, *V* = 0.91^2^, as well as for individual well-being, *F*(9, 243.52) = 2.04, *p* < 0.05, Wilks’ Λ = 0.84^3^.

The second step aimed at detecting if the four identity profiles differed for each dependent variables. A series of ANOVAs was performed. A Bonferroni correction was applied and the acceptable level of significance was fixed at *p* = 0.008 for collective well-being and at *p* = 0.017 for individual well-being. Results of ANOVAs are presented in Table 3. More specifically, results showed that profiles differed significantly on all but one indicator of well-being. Specifically, considering the Bonferroni correction, the impact of identity profiles belonging on life satisfaction was only marginally significant, *F*(3, 102) = 2.78, *p* = 0.045.

**Table 3:** **Means and ANOVAS’ results for the study variables as a function of clusters**.

Clusters	Cluster 1 – incoherent « original group » high	Cluster 2 – coherent strong identification	Cluster 3 – incoherent « Quebecers » high	Cluster 4 – incoherent « original group » low	*F*
**COLLECTIVE WELL-BEING INDICATORS**					
Collective esteem – Quebecers	6.01_a_	8.68_b_	7.58_b_	5.81_a_	19.04***
Collective hope – Quebecers	6.67_a_	8.42_b_	6.89_a_	5.71_a_	8.57***
Collective esteem – Canadians	7.05_a_	9.23_b_	7.42_a_	6.58_a_	10.26***
Collective hope – Canadians	7.38_a_	8.45_b_	7.20_a_	6.67_a_	5.34**
Collective esteem – original group	8.07_a_	8.35_a_	6.60_b_	5.27_b_	17.83***
Collective hope – original group	6.96	8.07_a_	6.42_b_	5.43_b_	5.74***
**INDIVIDUAL WELL-BEING INDICATORS**					
Self-esteem	8.28_a_	8.64_a_	8.05	6.91_b_	4.78**
Life satisfaction	6.91	7.49_a_	6.79	5.97_b_	2.78^†^
Subjective vitality	7.21	7.85_a_	6.77	6.23_b_	3.77*

*p < 0.017, **p < 0.008, ***p < 0.001, ^†^*p* < 0.05, non-significant due to Bonferonni correction.

*For each dependent variable, means with different subscripts indicate a significant difference at *p* < 0.05 using Scheffé’s *post hoc* tests*.

The third steps aimed at testing each of our hypotheses. To do so, Scheffe’s *post hoc* tests were conducted for each ANOVA to pinpoint specific profiles that differed on each specific indicator of well-being. Results of Scheffe’s *post hoc* tests (see Table 3) will be presented in relation with each of our hypotheses.

##### Hypothesis 1: comparing coherent and incoherent profiles

Based on the cognitive-development model of social identities integration (Amiot et al., 2007), it was first hypothesized that coherent profiles (Cluster 2) would be linked to higher levels of well-being than incoherent profiles (Cluster 1, 3, and 4; H1). Comparing the “Coherent – Strong identification” profile to the three other profiles, we can see that immigrants with a coherent identity displayed significantly higher scores on all indicators of collective and individual well-being than immigrants belonging to incoherent identity profiles. For example, the levels of collective hope for Quebecers (*M* = 8.42) and for Canadians (*M* = 8.45) of immigrants in the “Coherent – Strong identification” profile were significantly higher than the ones in the “Incoherent – Original group high” (Quebecers, *M* = 6.67; Canadians, *M* = 7.38), the “Incoherent – Quebecers high” (Quebecers, *M* = 6.89; Canadians, *M* = 7.20), and the “Incoherent – Original group low” (Quebecers, *M* = 5.71; Canadians, *M* = 6.67) profiles. The collective hope for the original group of immigrants in the “Coherent – Strong identification” profile (*M* = 8.07) was also significantly higher than the one of immigrants in the “Incoherent –– Quebecers high” (*M* = 6.42) and in the “Incoherent – Original group low” (*M* = 5.43) profiles. Similar patterns of results were obtained with collective esteem (see Table 3). Finally, immigrants in the “Coherent – Strong identification” profile also showed significant higher levels of self-esteem (*M* = 8.64), life satisfaction (*M* = 7.49), and subjective vitality (*M* = 7.85) than immigrants in the “Incoherent – Original group low” profile (self-esteem: *M* = 6.91; life satisfaction: *M* = 5.97; subjective vitality: *M* = 6.23). However, since immigrants in the coherent profiles also strongly identified to all groups, it seems premature to conclude that the obtained results are strictly due to coherence and not to a strong identification to all groups of belonging.

##### Hypothesis 2: impact of identification level among coherent profiles

As expected, profiles that naturally emerged from our data make it impossible to test the hypothesis stipulating that among coherent profiles, the ones where the strength of identification to the groups is highest would also display higher levels of well-being (H2). Indeed, since only one coherent profile emerged, with high levels of identification to the three groups, it cannot be compared to coherent profiles with lower levels of identification.

##### Hypothesis 3: impact of the dominant identity among incoherent profiles

We had two contradictory hypotheses concerning the impact of the dominant identity among incoherent profiles on immigrants’ well-being. First, based on the common ingroup identity model (Gaertner et al., 1993), it was hypothesized that immigrants’ well-being would be higher when immigrants strongly identified with the most inclusive group, namely the Canadians (H3a). Based on past research that highlighted the crucial role of the maintenance of the identification to the original group (Hornsey and Hogg, 2000a,b,c), it was also hypothesized that profiles where immigrants strongly identified with their original group would be associated to higher levels of well-being (H3b).

It seems impossible to argue on the importance of strongly identifying with a more inclusive group (H3a) since, as expected, none of the identity profiles that emerged had the “Canadians” identity as the only dominant identity. However, the level of identification to the original group seemed crucial for immigrants’ collective and individual well-being, thereby confirming H3b. Indeed, individuals in the “Incoherent – Original group low” profile displayed significantly lower level of collective esteem for their original group (*M* = 5.27) and of self-esteem (*M* = 6.91) than individuals in the “Incoherent – Original group high” profile (collective esteem for original group: *M* = 8.07; self-esteem: *M* = 8.28). Also, it is interesting to note that while well-being indicators such as collective hope for the original group (*M* = 5.43), life satisfaction (*M* = 5.97), and subjective vitality (*M* = 6.23) of immigrants in the “Incoherent – Original group low” profile are significantly lower than the ones of members of the “Coherent – Strong identification” profile (collective hope for original group: *M* = 8.07; life satisfaction: *M* = 7.49; subjective vitality: *M* = 7.85), the means of immigrants from the “Incoherent – Original group high” profile are not (collective hope for original group: *M* = 8.07; life satisfaction: *M* = 6.91; subjective vitality: *M* = 7.21). It thus seems that keeping a strong identification to the original group partially protects immigrants against some of the negative impacts of having an incoherent identity.

### Additional analyses

The BII model (Benet-Martinez et al., 2002), which constitutes another important model dealing with identity integration, was not included in the main part of the present paper because it does not directly tackle the issue of the optimal identity structure. Indeed, instead of focusing on the importance that should be allocated to each of the cultural groups of belonging, the BII is interested in the way people negotiate the belonging to two different cultural groups by addressing the concepts of perceived distance and conflicts between cultures. Because the BII constitutes an important model, supplementary analyses were conducted to see how perceived conflicts and distance between the original group’s and Quebecer’s and Canadian’s cultures were associated with the well-being of Canadians immigrants living in the province of Quebec. This also allowed a comparison between the relevance of identity profiles and of perceived conflicts and distance between identities to predict immigrants’ well-being.

Nine multiple regressions, one for each indicator of collective and individual well-being, were conducted. Immigrants’ perception of distance and conflict between Quebecers and their original group, and between Canadians and their original group were entered simultaneously as predictors of each well-being indicator. Results are presented in Table 4. In brief, while perceived distance between identities was negatively related to only a few indicators of collective well-being, perceived conflicts did not seem to be an important predictor of collective well-being. Surprisingly, a *positive* link was even found between conflicts between the Quebecer’s and the original group’s cultures and collective hope for Quebecers. In addition, the two concepts proposed in the BII were not related to individual well-being. Results obtained with the BII are thus inconsistent and do not seem to capture factors that are crucial for the well-being of Canadian immigrants living in the province of Quebec.

**Table 4:** **Immigrants’ well-being as a function of perceived distance and conflict between cultures**.

	Distance – Quebecer and original group cultures		Conflict – Quebecer and original group cultures		Distance – Canadian and original group cultures		Conflict– Canadian and original group cultures		*R*	*F*	*p*
	*B*	*p*	*B*	*p*	*B*	*p*	*B*	*p*			
**COLLECTIVE WELL-BEING INDICATORS**											
1. Collective esteem – Quebecers	−0.35	<0.001	0.15	0.33	0.15	0.10	−0.12	0.40	0.38	4.55	<0.01
2. Collective hope – Quebecers	−0.25	<0.01	0.39	<0.05	0.12	0.19	−0.22	0.13	0.38	4.38	<0.01
3. Collective esteem – Canadians	0.06	0.53	−0.04	0.79	−0.35	<0.001	−0.07	0.61	0.41	5.46	<0.001
4. Collective hope – Canadians	0.10	0.19	0.09	0.47	−0.18	<0.05	−0.05	0.70	0.24	1.59	0.18
5. Collective esteem – original group	−0.02	0.85	0.13	0.43	−0.23	<0.05	−0.10	0.54	0.32	2.95	<0.05
6. Collective hope – original group	−0.15	0.15	0.03	0.89	−0.07	0.52	0.08	0.65	0.30	2.50	<0.05
**INDIVIDUAL WELL-BEING INDICATORS**											
7. Self-esteem	0.01	0.87	0.12	0.37	−0.12	0.12	−0.21	0.09	0.25	1.63	0.17
8. Life satisfaction	−0.10	0.24	−0.01	0.93	−0.06	0.47	−0.24	0.07	0.35	3.53	<0.01
9. Subjective vitality	0.00	0.96	0.14	0.31	−0.09	0.27	−0.27	<0.05	0.25	1.69	0.16

## Discussion

Migration between countries is a worldwide phenomenon that generates greater cultural diversity within nations. Consequently, a growing number of people have to integrate multiple cultural identities within their global self-concept. This increased cultural diversity highlights the need for a better understanding of the role that cultural affiliations plays in people’s well-being.

The present study aimed at better understanding the particular situation of Canadian immigrants living in the province of Quebec in order to provide a more accurate picture of how multiple cultural belonging affects immigrants’ well-being. More specifically, the main goal of the study was to pinpoint the organization of multiple cultural identities that maximizes the well-being of Canadian immigrants living in Quebec. Results highlighted the importance of achieving coherence between identities, while maintaining a strong identification to the original cultural group. The situation of Quebecer immigrants is particular in many ways. To begin, two of the identities that they have to integrate (Quebecer and Canadian) might be perceived as conflictual by many people. This might explained why the Canadian identity, which was also considered the more inclusive one, did not play a crucial role in immigrants’ well-being. Immigrants might indeed feel that they have to choose between the Canadian and the Quebecer identities and thereby benefit from identifying to the one that they come the most in contact with everyday, namely the Quebecer culture. It is therefore possible that studying Canadian immigrants living in another province such as British Colombia might have yielded different results that could have, for example, been in favor of the common ingroup identity model (Gaertner et al., 1993). Considering that researchers have recently started to emphasize the importance of the host society in immigrants’ acculturation process (Van Oudenhoven et al., 2006), it seems plausible that factors such as Canadians’ high level of acceptance of multiculturalism (Berry and Kalin, 1995; Guimond et al., in press) might play a role in the identity structure that should be favored by Canadian immigrants. Indeed, it might be easier and more beneficial for Canadian immigrants to achieve coherence between identities and to maintain their original culture than it is for immigrants moving to countries with other national policies than multiculturalism, such as colorblindness in France (based on decategorization; racial, or ethnic membership should not matter because people are all the same; Richeson and Nussbaum, 2004) or assimilation in Germany (immigrants are expected to adopt the culture of the dominant group and leave behind their own cultural characteristics; Taylor and Moghaddam, 1994). Past research has shown that such national policies do have an impact on intergroup attitudes (Guimond et al., in press), which in turn influence immigrants’ integration process.

The present research, driven by the desire to integrate cultural and clinical psychology, leads to four main contributions that have both theoretical and practical implications. The first contribution is that it allows a better understanding of immigrants’ integration process. Authors in the field do not agree on the importance that should be given to each cultural identity in an integrated self-concept. Does having an integrated identity mean strongly identifying to all groups of belonging? Does it mean to be able to equally and simultaneously identify to all groups? Or does it mean to come to identify to a group that includes all groups of belonging? Our study allowed to contrast existing theories and to simultaneously compare the role played by coherence, strengths of identification, and identification to a more inclusive group in immigrants’ well-being. In line with the cognitive-development model of social identity integration (Amiot et al., 2007) and with preliminary empirical results pertaining to this model (de la Sablonnière et al., 2010), our findings indicate that coherence between identities seems crucial for the well-being of Quebecer immigrants. Indeed, results revealed that immigrants with a coherent identity profile (i.e., “Coherent – Strong identification”) reported higher levels of collective and individual well-being than those belonging to the three other incoherent profiles. However, since our data did not allow to test whether coherent profiles with lower levels of identification would also be linked to higher levels of well-being, one might ask whether the present results are an artifact of all levels of identification in the “Coherent – Strong identification” profile being high, such as proposed by the acculturation model (Berry, 1997). To answer this question, we can compare the “Incoherent – Quebecers high” and the “Coherent – Strong identification” profiles. Immigrants from the incoherent profile identify almost as strongly to the three groups as immigrants from the coherent profile (see Table 2). The only difference between these two groups lies is the identification to Canadians, which is much lower. Considering that identification to Canadians does not seem to be a key factor for immigrants’ well-being in our study, it can be argued that the difference between these two profiles is mainly in terms of coherence between identities. Yet, immigrants from the coherent profile display higher levels of collective well-being. Such findings, combined to past studies with bicultural immigrants that showed that coherence between identities is a better predictor of well-being than the strength of identification to the groups (de la Sablonnière et al., 2010), contribute to the literature by guiding researchers toward a unique definition of identity integration, and clinicians toward a clear goal: the one of helping their clients to attain coherence between their multiple identities.

The second contribution of our study, in line with the work of Hornsey and Hogg (2000a,b,c), pertains to demonstrating the key role played by a strong identification to the original cultural group in immigrants’ individual well-being. Indeed, belonging to the “Incoherent – Original group high” profile, as contrasted to the “Incoherent – Original group low” profile, had positive impacts on some indicators of original group’s collective well-being (collective hope and collective esteem), as well as on personal well-being (self-esteem). Results also suggested that, even though coherence between the strengths of identification is linked to better outcomes, maintaining a strong identification to the original group seems to protect against the negative impact of incoherence on life satisfaction and subjective vitality. Once again, the fact that Canada’s national policies enable the maintenance of immigrants’ culture probably contributes to the positive link found between the maintenance of a strong identification to the original group and Quebecers immigrants’ well-being.

Thirdly, the present study contributes to the understanding of immigrants’ well-being over and above what was already offered by important existing models. For instance, ANCOVAs were conducted to see if the impact of identity profiles on well-being remained the same, even when controlling for two important concepts in the literature, namely perceived conflicts and distance between cultures (Benet-Martinez et al., 2002). Results pertaining to the link between identity profiles belonging and the various well-being indicators did remain the same, thereby confirming the relevance of studying identity profiles in order to find strategies to maximize the well-being of Canadian immigrants living in the province of Quebec. These results are in line with a recent study that showed that coherence between identities better predicts immigrants’ well-being than distance and conflicts between identities (de la Sablonnière et al., 2010).

Finally, the last main contribution of this study is that it employs a methodology that can also be used by clinicians to assess their clients’ identity structure, thereby allowing them to better target their interventions. We now know that, in order to picture the identity profile of their clients and then guide them better, clinicians should explore questions such as: to which cultural referents does the client relate? Does he feel that he belongs to one or several cultural groups? How strongly does he relate to each of these groups? Up to now, researchers have theoretically proposed various ways of organizing multiple cultural identities and then empirically tested their propositions by forcing participants into pre-established groups. These theoretical models require strict methodological and statistical approaches to be tested, requirements that are not always necessarily met (see Rudmin, 2003). Instead, the present study searched for identity structures that were naturally present in an immigrants’ sample and confirmed that these structures are related to differences in immigrants’ well-being. Using identity profiles thereby offers more flexibility, assures that the proposed structures are really present in a population of immigrants and can easily be used by clinicians, while ensuring a strong predictive power of immigrants’ well-being.

Despite the theoretical and applied contributions of the present study to the fields of cultural and clinical psychology, a few limitations need to be mentioned. First, our study was interested in a really specific category of immigrants, namely Canadian immigrants living in the province of Quebec, making it hard to generalize our results to all multicultural individuals. Future studies should aim at replicating our results with sample of Canadian immigrants from other provinces, or with immigrants from a host society that has immigration policies that differ from the “multiculturalism” one of Canada.

Second, although we controlled for variables such as the country of origin and the number of years since arrival in Canada, other inter-individual differences (i.e., religion, status of the original group, mother tongue) should be taken into consideration in future studies. Indeed, in the present research, immigrants were examined as a single group even though they came from different contexts, which might have an influence either on identity profiles belonging or might act as a moderator of the impact of identity profiles on well-being. For exploratory purposes, the moderating impact of the status of the country of origin on the impact of identity profiles on well-being indicators was tested. The Human Development Index from the United Nations Development Programme (2011) was used to evaluate the status of the different countries. No moderating effect was found, except for the impact of identity profiles on self-esteem. Results indicated that while the “Incoherent – Original group high” and “Coherent – Strong identification” profiles generally maximize immigrants’ self-esteem, only maintaining a strong identification to the original group allows a stronger self-esteem when the status of the original group is low. Including additional inter-personal factors in future studies might offer a more complete picture of the optimal identity structure.

The third limitation of the present study is that participants were limited to three groups of belonging. Indeed, participants were asked to indicate the group they considered their cultural group of origin and this group was used as their “third identity”. However, some participants might have had more complex ethnic backgrounds, leading them to identify with more than one cultural group prior to their arrival in Canada. Even though our methodology allowed drawing a more complete picture of people’s organization of multiple cultural identities than past studies that considered only two cultural groups of belonging (i.e., culture of origin and culture of the society of settlement), qualitative studies could for example be used in the future to study the optimal identity structure of immigrants with more complex cultural backgrounds.

Fourth, it is important to underline that some indicators of individual well-being can be considered problematic from a cultural standpoint. Heine and Hamamura (2007) for example showed that East Asians generally report lower levels of self-esteem than Westerners. Research also showed that these self-critical attitudes were less distressful for Japanese than for European or Asian Canadians, being more weakly related to depression scores in the former than in the latter population (Heine and Lehman, 1999). The authors explained these results by the fact that Westerners tend to be more concerned about maintaining self-esteem, whereas East Asians tend to be more focused on maintaining face, i.e., how they are being evaluated by others (Heine, 2004). Important differences across nations in their mean levels of life satisfaction were also consistently found (Veenhoven, 1993; Diener et al., 2003). Differences in nations’ average level of income (Diener et al., 1995) or Westerners’ tendency to self-enhanced (Heine et al., 2000) were proposed as potential causes for the observed differences in life satisfaction. The fact that self-esteem and life satisfaction measures seem to be culturally biased raises doubts about the validity of these measures when studying the well-being of immigrants. However, since similar results were obtained with indicators of both individual and collective well-being in our study, results obtained with indicators of individual well-being can be seen as complementary to the ones obtained with collective well-being indicators. To overcome this limitation, future research could also include mental health indicators, such as measures of anxiety, depression, or suicidal ideation, or indicators of physical well-being, such as blood pressure or salivary cortisol, to provide a better picture of the potential consequences of different identity configurations.

Finally, two additional limitations are also worth mentioning. First, the fact that participants were recruited in French classes might have introduced a selection bias. Indeed, immigrants engaged in such classes may be those who are most motivated to integrate the cultures of their society of settlement, thereby contributing to the crucial role played by coherence in immigrants’ well-being. It might be that, being initially motivated to integrate the Quebecer and Canadian cultures, the more they succeed at it, the more their well-being benefits from it. The present study needs to be replicated to see if similar results would be obtained with immigrants recruited in other contexts. Second the present study employed a correlational design, which makes causality inferences between identity profiles belonging and well-being impossible. One may for example argue that well-being do not improve because of change in identity structure, but that it is immigrants with greater well-being that have a tendency to organize their identity differently. Future research should replicate these findings using an experimental design where identification to certain groups would be manipulated, or a longitudinal design that links changes in identity structure to changes in well-being.

Considering the growing number of immigrants worldwide, the proportion of the psychologists’ clientele being born outside of their country will not cease to increase in the future. A better understanding of immigrants’ particular challenges is therefore helpful in clinicians’ day-to-day practice. Prior to our research, it would have been hard for clinicians to know how to deal with the identity challenges faced by their multicultural clients. Based on our findings, clinicians dealing with clients whose psychological well-being suffers from migration may: (1) investigate identification with the original cultural group (e.g., Has it been maintained? Is it possible to integrate this culture in their life in their new culture?); (2) explore if identification with the original cultural group is in harmony with other cultural groups of belonging (e.g., Is it possible to simultaneously identify to their multiple cultural groups of belonging? Are there conflicts between the various identities?); and (3) as proposed by Amiot et al. (2007), work with their clients on finding links and connections between their multiple groups of belonging to help them identify with the new groups, without impeding identification to the original group. Techniques such as focusing on similarities between groups, or writing about conflicting identities, have been shown to be efficient ways to increase levels of identity integration and well-being (Huberdeau et al., in preparation).

In conclusion, the present research offers compelling insights on the way cultural identity, and mainly multiple cultural identities’ organization, can affect immigrants’ well-being. It offers concrete and new avenues to help clinicians working with multicultural individuals, while offering cultural psychology an improved perspective of identity integration. Considering that cultural diversity is omnipresent in today’s world and that it will only increase with time, the need for studies that integrate the knowledge of cultural and clinical psychologists will only become greater in years to come.

## Conflict of Interest Statement

The authors declare that the research was conducted in the absence of any commercial or financial relationships that could be construed as a potential conflict of interest.

## Footnotes

^1^In the present article, the term “Quebecers” will refer only to the French-speaking inhabitants of the province of Quebec, although it theoretically refers to all of Quebec residents. Yet, we chose this term to refer to the culture associated with the francophone culture in Quebec because it is how the majority of people in Canada refer to it.

^2^The Pillai–Barrett’s trace was used because this indicator is the more robust when the Box’s test indicates that covariance matrices are not homogeneous, *F*(18, 12304.49) = 2.62, *p* < 0.001, *M* = 50.66, while groups’ sizes are unequal and the greatest covariance is in the smallest group (Tabachnick and Fidell, 2007).

^3^The Wilks’ Lambda was used because the Box’s test confirmed that covariance matrices are homogeneous, *F*(18, 12304.49) = 1.13, *p* = 0.32, *M* = 21.77.
